# Environmental Context-Aware Human Action Recognition from 4D Millimeter-Wave Radar Point Clouds

**DOI:** 10.3390/s26185717

**Published:** 2026-09-09

**Authors:** Xiaozhi Li, Zhipeng Lou, Wei Liang, Chao Zhou

**Affiliations:** 1 Radar Technology Research Institute, School of Information and Electronics, Beijing Institute of Technology, Beijing 100081, China; lixz@bit.edu.cn; 2 School of Computer Science and Technology, Beijing Institute of Technology, Beijing 100081, China; 3120230908@bit.edu.cn (Z.L.); liangwei@bit.edu.cn (W.L.); 3 Innovative Equipment Research Institute, Beijing Institute of Technology, Chengdu 610299, China

**Keywords:** 4D millimeter-wave radar, human action recognition, indoor scene sensing, environmental context, floorplan inversion

## Abstract

As an emerging sensing modality, 4D millimeter-wave radar provides a privacy-preserving and lighting-robust solution for indoor scene perception and human action recognition (HAR). However, existing radar-based HAR methods mainly focus on short-term motion dynamics while overlooking the influence of indoor scene context. Furthermore, reconstructing indoor layouts from sparse and irregular radar point clouds remains challenging. To address these issues, this paper proposes an environmental context-aware radar action recognition framework (ECRAR) that jointly models human motion and indoor scene context from 4D millimeter-wave radar point clouds. Specifically, a trajectory-to-floorplan inversion branch reconstructs indoor layouts from long-term trajectory point clouds by exploiting the relationship between human movement distributions and spatial accessibility. A motion feature extraction branch captures discriminative short-term motion representations through multi-scale spatial modeling, while an environmental context fusion module adaptively integrates scene and motion features using cross-attention. We also construct InActivity-Scene, a new dataset with fine-grained scene annotations and nine categories of daily and hazardous human actions collected in multiple indoor environments. Experimental results demonstrate that ECRAR consistently outperforms existing methods, achieving 92.2% recognition accuracy with significant improvements on environment-dependent actions, while ablation studies further verify the effectiveness of each proposed component.

## 1. Introduction

Human action recognition (HAR) is a fundamental task in intelligent sensing systems, with broad applications in healthcare monitoring, smart homes, elderly care, and human–computer interaction [1]. Among various sensing modalities, 4D millimeter-wave (mmWave) radar, as an active microwave sensing technique, has attracted increasing attention for its privacy preservation, low sensitivity to illumination, and strong robustness against occlusion. Compared with vision-based approaches, mmWave radar avoids capturing sensitive visual information while maintaining stable perception in complex indoor environments [2].

With the rapid development of radar sensing hardware, recent studies have increasingly adopted point cloud representations generated by mmWave radar for HAR. Existing methods commonly extract spatio-temporal features from radar point cloud sequences using convolutional neural networks, recurrent neural networks, or Transformer-based architectures [3,4]. Although these methods have achieved promising performance, most of them focus primarily on short-term motion patterns and treat the surrounding environment as irrelevant background [5,6]. However, human actions are inherently constrained and supported by indoor scene layouts and object distributions. For example, sitting, lying down, and standing up usually occur near interactive objects such as chairs, sofas, or beds, whereas walking trajectories are constrained by accessible spatial regions. Ignoring such environmental context may lead to ambiguities between actions with similar motion patterns but different scene dependencies.

A key challenge in context-aware radar HAR is how to obtain useful indoor environmental information from radar observations. Conventional radar signal processing pipelines commonly suppress static clutter to emphasize moving targets, which may discard structural scene information [7]. Meanwhile, directly reconstructing indoor layouts from sparse and irregular radar point clouds remains difficult because radar observations are incomplete, discontinuous, and affected by motion uncertainty. Nevertheless, long-term human trajectories provide implicit evidence of spatial accessibility and functional usage. Frequently visited regions usually correspond to walkable or interactive areas, whereas rarely visited regions may indicate obstacles, walls, or inaccessible zones. This observation suggests that human trajectory distributions can be exploited for indoor scene sensing and floorplan-level environmental perception.

Motivated by this, we propose an environmental context-aware radar action recognition framework (ECRAR) for indoor scene sensing and HAR from 4D mmWave radar point clouds. The framework jointly models long-term indoor scene context and short-term human motion dynamics. Specifically, a trajectory-to-floorplan inversion branch is designed to recover structured indoor scene layouts from long-term human trajectory point clouds by exploiting the relationship between movement distributions and spatial accessibility. In parallel, a motion feature extraction branch learns discriminative short-term motion representations from radar point cloud sequences through multi-scale spatial modeling. Finally, an environmental context fusion module adaptively integrates scene and motion features via a cross-attention mechanism, enabling the model to capture human–environment interactions for context-aware HAR.

To support research on scene-aware radar HAR, we further construct a new dataset named InActivity-Scene. Unlike existing radar HAR datasets that mainly provide action labels, InActivity-Scene contains fine-grained scene annotations, object boundary information, and 4D radar point cloud sequences collected across multiple real indoor environments. The dataset includes nine categories of human actions in bedrooms, living rooms, bathrooms, and kitchens, providing diverse environmental contexts for evaluating indoor scene sensing and action recognition methods.

The main contributions of this paper are summarized as follows:

1. A context-aware radar-based human action recognition framework is proposed to jointly model human motion dynamics and indoor environmental context from 4D mmWave radar point clouds.
2. A trajectory-to-floorplan inversion method is introduced to recover indoor scene layouts from long-term human trajectory point clouds, enabling floorplan-level environmental perception without additional sensing devices.
3. A new scene-aware radar HAR dataset, InActivity-Scene, is constructed with fine-grained environmental annotations and diverse real indoor scenarios.

The remainder of this paper is organized as follows. Section 2 reviews related work. Section 3 presents the proposed methodology. Section 4 introduces the InActivity-Scene dataset. Section 5 reports the experimental settings, comparative results, and ablation studies. Section 6 discusses the experimental findings, implications, limitations, and future research directions. Finally, Section 7 concludes the paper.

## 2. Related Work

Recent studies on human action recognition (HAR) can be broadly categorized into vision-based sensing, non-vision sensing, and radar-based sensing. Vision-based approaches achieve remarkable recognition accuracy but suffer from privacy concerns and sensitivity to illumination variations. Non-vision sensing methods, including wearable sensors and wireless signals, improve privacy preservation but are constrained by device dependence or limited environmental perception. More recently, 4D mmWave radar has attracted increasing attention because it provides robust motion perception while preserving user privacy. The following sections review representative studies in these categories and discuss their limitations, which motivate the proposed environmental context-aware radar HAR framework.

### 2.1. Vision-Based HAR

Vision-based HAR has been extensively studied using RGB and depth cameras and remains one of the most mature research directions. Existing methods mainly rely on deep learning architectures, including convolutional neural networks (CNNs), recurrent neural networks (RNNs), and Transformers, to jointly model spatial appearance and temporal dynamics from visual inputs.

CNN-based methods have proven effective at learning spatial representations from image sequences. Representative works include two-stream networks that separately extract spatial and temporal features from RGB frames and optical flow [8], as well as subsequent improvements in spatiotemporal feature fusion [9]. To better capture motion at different temporal resolutions, SlowFast networks process low- and high-frame-rate video streams simultaneously [10], while multi-stream architectures further incorporate motion saliency to improve action discrimination [11]. In addition, 3D convolutional networks have been adopted to learn spatial and temporal representations [12], with further extensions improving robustness to action speed variations [13] and viewpoint changes [14].

RNN-based methods provide an effective solution for modeling temporal dependencies in human actions. Representative architectures, such as Long Short-Term Memory (LSTM) and Gated Recurrent Unit (GRU) networks, have demonstrated competitive performance in HAR by effectively capturing sequential motion patterns [15,16,17]. More recently, Transformer-based methods have become increasingly popular because self-attention mechanisms enable global temporal modeling over long video sequences. Representative methods include Action Transformer [18], which combines object detection with self-attention for action localization, and VidTr [19], which aggregates spatiotemporal information through stacked attention modules for video classification.

Although vision-based methods have achieved outstanding recognition accuracy, their performance is highly dependent on lighting conditions and camera viewpoints. More importantly, camera-based sensing inevitably raises privacy concerns, making it less suitable for long-term monitoring in indoor environments such as homes, hospitals, and elderly care facilities.

### 2.2. Non-Vision Based HAR

To address the limitations of vision-based sensing, considerable efforts have been devoted to non-vision HAR owing to its superior privacy preservation and robustness to illumination variations. Existing non-vision approaches can be broadly divided into wearable sensor-based and wireless signal-based methods. Unlike camera-based systems, these methods do not rely on visual observations and therefore provide a more privacy-preserving solution for long-term indoor monitoring.

Wearable sensor-based HAR utilizes inertial sensors such as accelerometers and gyroscopes to capture body motion signals. Early studies primarily relied on handcrafted statistical features combined with conventional machine learning classifiers. More recently, deep learning models have become the dominant solution by directly learning discriminative temporal representations from raw sensor signals. Representative approaches employ residual convolutional networks with adaptive denoising mechanisms to improve feature robustness under noisy sensing conditions [20], multi-branch CNN-BiLSTM-BiGRU hybrid architectures to jointly exploit local motion patterns and long-term temporal dependencies [21], and hierarchical CNN-LSTM frameworks for temporal feature learning [22]. Despite their promising recognition accuracy, wearable sensing methods require users to carry or wear dedicated devices, reducing user comfort and limiting their applicability in long-term unattended monitoring. Moreover, wearable sensors mainly capture body motion and lack the ability to capture global environmental context, making it difficult to model interactions between human actions and indoor scenes.

Wireless signal-based HAR enables device-free sensing by exploiting variations in WiFi Channel State Information (CSI) caused by human movements. Representative methods include cross-environment feature learning frameworks [23], autoencoder-based CSI representation learning [24], and hybrid CNN-RNN architectures for joint spatial-temporal modeling [25]. Despite their advantages in privacy preservation and device-free sensing, WiFi-based HAR methods remain sensitive to environmental dynamics and signal propagation variations. Complex indoor layouts, multipath interference, and changes in surrounding objects may significantly affect signal stability and recognition performance. Moreover, WiFi sensing methods generally provide limited spatial structural information, making it difficult to explicitly model human–environment interactions.

### 2.3. Radar-Based HAR

Millimeter-wave radar has emerged as a promising sensing modality for HAR because it provides robust motion perception under poor illumination while naturally preserving user privacy. Compared with conventional wireless sensing, radar measurements additionally provide explicit spatial information, enabling more fine-grained analysis of human movements and the surrounding indoor environment.

Early radar-based HAR mainly relied on 3D mmWave radar, where micro-Doppler signatures or time-frequency spectrograms served as the primary motion representations. Representative works combine time-frequency spectrograms with BiLSTMs to jointly model spatial and temporal features [26], introduce attention mechanisms to enhance discriminative micro-Doppler features [27], and employ convolutional temporal autoencoders with perceptual loss for abnormal gait recognition [28]. Although these methods achieved promising performance, they mainly depended on handcrafted radar representations and lacked explicit three-dimensional spatial perception.

The emergence of 4D mmWave radar has substantially improved radar sensing by providing elevation measurements in addition to range, azimuth, and Doppler information. Consequently, radar point clouds have become the dominant data representation for HAR, enabling more accurate modeling of human posture and spatial motion patterns. Existing studies generally follow two technical routes.

The first category converts radar point clouds into regular voxel-based or grid-based representations for feature extraction. Representative works improve radar-based HAR through spatiotemporal feature learning, person-action modeling, and voxelized point-cloud processing [29,30,31]. Recent studies have further focused on improving the efficiency and representation capability of voxel-based HAR, including voxel preprocessing with multi-view occupancy-gated convolution for sparse point-cloud feature extraction [32], as well as early spatiotemporal fusion on voxelized radar point clouds using compact 3D–2D hybrid networks [5].

The second category directly processes raw radar point clouds to better preserve their geometric information. Representative works leverage point-based architectures to extract discriminative spatiotemporal features directly from sparse point clouds [33,34,35]. Recent studies have further explored advanced point-cloud architectures for more effective spatial and temporal modeling, including spatial–temporal Point Mamba architectures for jointly capturing geometric structures and long-range temporal dependencies [36], as well as multi-frame fusion and stochastic sampling strategies combined with attention-enhanced temporal modeling to alleviate point-cloud sparsity [6]. Graph-based methods have also shown strong capability in exploiting the geometric relationships of sparse radar point clouds for action recognition [37]. More recent graph-based approaches further construct star-graph representations of variable-size radar point-cloud sequences and employ discrete dynamic graph neural networks to jointly model intra-frame and inter-frame spatial–temporal relationships [38].

Overall, recent advances in radar-based HAR have significantly enhanced the spatial representation and spatiotemporal modeling of radar point-cloud sequences through increasingly advanced voxel-based, point-based, and graph-based architectures. Nevertheless, despite these advances in motion representation and spatiotemporal modeling, existing methods primarily concentrate on human motion itself while largely neglecting the surrounding environmental context and its relationship with human activities. In realistic indoor scenarios, human actions are closely associated with scene layouts and object distributions, which provide valuable semantic cues for distinguishing actions with similar motion patterns. Motivated by this observation, the proposed method recovers indoor scene structures from long-term radar trajectories and integrates environmental context with motion features to achieve context-aware human action recognition.

## 3. Methodology

### 3.1. Overview of the ECRAR

**Figure 1:**
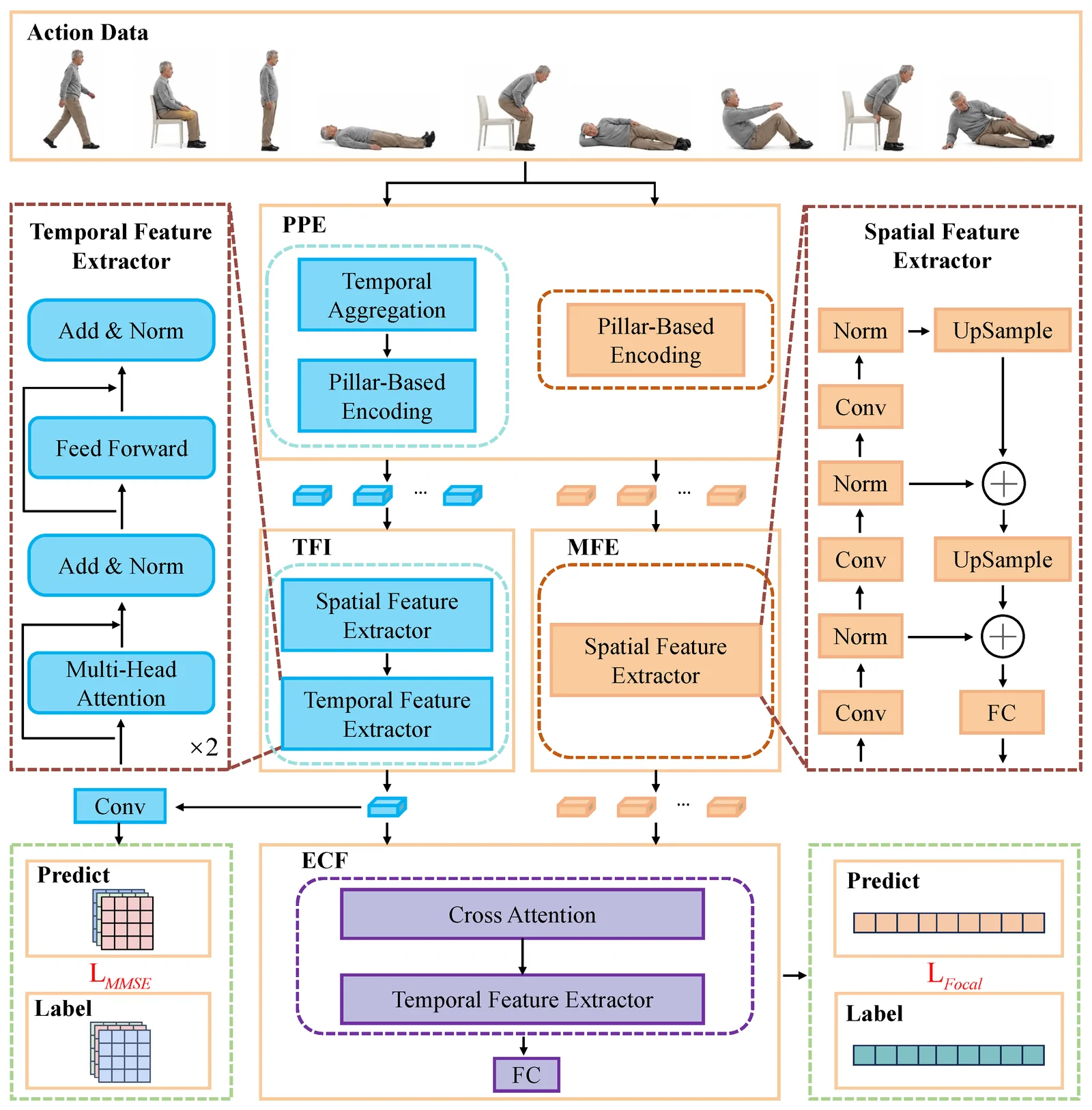
Architecture of the proposed ECRAR framework.

To incorporate scene awareness into radar-based HAR, we propose ECRAR, an environmental context-aware 4D mmWave radar human action recognition framework. The proposed framework jointly models human motion dynamics and indoor environmental structure, enabling environmental context-aware human activity recognition. The overall architecture is illustrated in Figure 1.

The ECRAR consists of four major components: point cloud preprocessing and pillar-based encoding (PPE), trajectory-to-floorplan inversion module (TFI), motion feature extraction module (MFE), and environmental context fusion module (ECF).

Specifically, the PPE module first performs temporal normalization, trajectory aggregation, and pillar-based voxelization on raw radar point cloud sequences, converting sparse and irregular radar observations into structured spatial representations suitable for subsequent feature learning. Based on the encoded pillar representations, the TFI module utilizes long-term human trajectory point clouds to reconstruct indoor environmental structures. Meanwhile, the MFE module extracts discriminative short-term motion features through multi-scale spatial modeling, enabling effective characterization of different human activity patterns. Finally, the ECF module adaptively integrates environmental context features with motion representations through a cross-attention mechanism. By dynamically associating human motion patterns with relevant environmental context, the proposed framework captures human–environment interactions and achieves context-aware human activity recognition.

### 3.2. Point Cloud Preprocessing and Pillar-Based Encoding

#### 3.2.1. Temporal Aggregation

The raw outputs of 4D mmWave radar consist of sparse point cloud sequences with varying temporal lengths and irregular frame distributions, which may introduce unstable optimization and feature inconsistency during training. Therefore, temporal normalization and aggregation are first performed to ensure consistent sequence representation and suppress trajectory noise.

For short-term action recognition, all radar sequences are normalized to a fixed temporal length *T*. Sequences shorter than *T* are padded with zero tensors, while binary masks are introduced to distinguish valid frames from padded frames. This strategy enables efficient batch-wise training while avoiding feature interference caused by invalid frames.

For long-term trajectory modeling, a one-minute trajectory window is constructed for each action sample. The proposed framework adopts a dual-timescale design: the short-term action sequence is used to characterize fine-grained motion dynamics for action recognition, whereas the long-term trajectory sequence characterizes the spatial distribution for environmental reconstruction. Therefore, the two inputs serve different functional roles in the proposed framework rather than simply providing action observations at different temporal lengths. Instead of directly processing all radar frames, point clouds within each second are aggregated into a single frame:

(1) $$P_{i}^{agg}=\underset{t\in \tau_{i}}{⋃}P_{t}$$

where $P_{t}$ denotes the radar point cloud at timestamp *t*, and $\tau_{i}$ represents the set of frames within the *i*-th second.

Since floorplan inversion mainly depends on long-term spatial coverage rather than short-term motion details, temporal aggregation improves trajectory continuity and spatial density, effectively mitigates the impact of point cloud sparsity and suppresses random noise. Moreover, second-level aggregation significantly reduces computational redundancy, thereby improving trajectory modeling efficiency. Accordingly, the environmental information contained in a long-term trajectory is primarily determined by its spatial coverage, while the temporal duration mainly affects the opportunity to observe a broader range of human movements.

#### 3.2.2. Pillar-Based Encoding

Radar point clouds are sparse, unordered, and irregular, making direct feature extraction computationally inefficient. Although conventional voxelization methods discretize the entire 3D space into regular voxels, most voxels remain empty due to the sparsity of radar point clouds, leading to substantial computational overhead. To improve efficiency while preserving structural information, a pillar-based voxelization strategy is adopted.

Instead of discretizing the entire 3D space, the proposed method partitions only the horizontal plane into H×W grid cells while retaining vertical point distributions within each pillar. This representation reduces computational redundancy while preserving posture-related vertical characteristics that are important for activity understanding. For each pillar, three statistical features are extracted:

(2) $$f_{i}=\left[h_{i}^{\max},h_{i}^{\min},n_{i}\right]$$

where $h_{i}^{\max}$ and $h_{i}^{\min}$ denote the maximum and minimum point heights, respectively, and $n_{i}$ represents the number of points within the pillar.

The height features of the point cloud characterize vertical posture distributions, while point density reflects local occupancy and motion intensity. These complementary features jointly provide compact yet effective geometric representations for both motion recognition and scene reconstruction. Finally, each radar frame is represented as:

(3) $$X\in R^{3\times H\times W}$$

which can be efficiently processed using convolutional neural networks.

**Figure 2:**
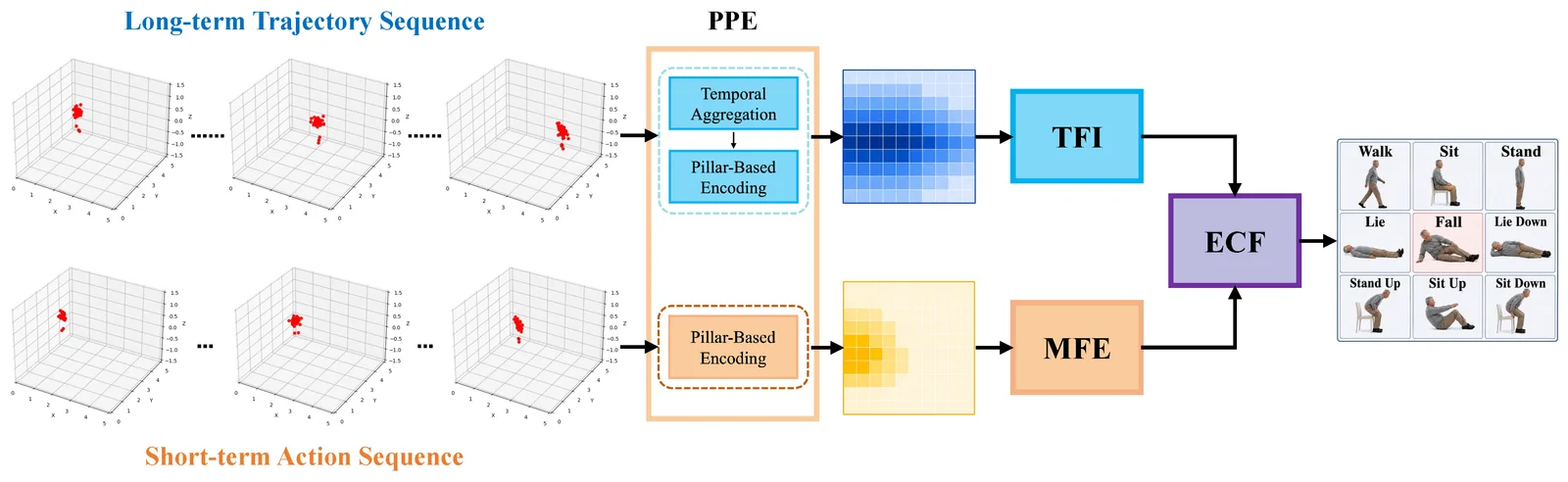
Point cloud processing and feature extraction in ECRAR.

Figure 2 provides a visual illustration of the intermediate point cloud processing and feature extraction stages for the long-term trajectory and short-term action sequences.

### 3.3. Trajectory-to-Floorplan Inversion Module

The trajectory-to-floorplan inversion module is one of the core contributions of this work. Unlike existing radar HAR methods that suppress static environmental information during preprocessing, the proposed method reconstructs indoor environmental context directly from long-term human trajectory point clouds.

Since human movement is constrained by indoor layouts, long-term trajectory distributions implicitly encode spatial accessibility and functional partitioning. Humans tend to exhibit distinct motion patterns across functional areas, enabling environmental structures to be inferred from trajectory distributions. To model the relationship between long-term trajectories and indoor layouts, an encoder–decoder architecture is designed. The TFI module receives only radar-observed trajectory information and is optimized for floorplan reconstruction. No action label or classification result of the corresponding action sample is directly provided to the TFI module. This reconstruction objective encourages the long-term trajectory representation to emphasize spatial accessibility and environmental structure rather than directly performing action prediction. Moreover, action predictions are not fed back to the TFI module or used to update the environmental representation, thereby avoiding error propagation from action classification to subsequent environmental context modeling.

#### 3.3.1. Spatial Feature Extractor

An FPN is employed to extract multi-scale spatial features from trajectory frames, enabling the model to capture both fine-grained accessibility patterns and large-scale scene structures. The encoded trajectory sequence is represented as:

(4) $${\hat{X}}^{tra}\in R^{T_{tra}\times d}$$

where ${\hat{X}}^{tra}$ denotes the spatially encoded trajectory features, $T_{tra}$ is the number of trajectory frames, and *d* is the feature dimension.

#### 3.3.2. Temporal Feature Extractor

To capture long-range temporal dependencies across the trajectory sequence, we employ a Transformer encoder for temporal modeling. Positional encoding is introduced to preserve temporal order information:

(5) $$Z^{tra}={\hat{X}}^{tra}+PE$$

where PE denotes positional encoding.

The Transformer encoder then performs self-attention and feed-forward transformation with residual connections and layer normalization:

(6) $$\begin{array}{cc} A^{tra} & =\mathrm{LayerNorm}\left(Z^{tra}+\mathrm{Attention}\left(Q,K,V\right)\right),\\ X_{out} & =\mathrm{LayerNorm}\left(A^{tra}+\mathrm{FFN}\left(A^{tra}\right)\right), \end{array}$$

where Attention(Q,K,V) denotes the standard multi-head self-attention operation, with *Q*, *K*, and *V* representing the query, key, and value matrices derived from $Z^{tra}$, respectively. LayerNorm(·) denotes layer normalization, and FFN(·) denotes the feed-forward network.

Self-attention enables the model to globally aggregate trajectory information and dynamically emphasize trajectory observations that are more informative for floorplan reconstruction. After Transformer encoding, the output sequence is represented as:

(7) $$X_{out}=\left\{X_{1}^{out},X_{2}^{out},\ldots ,X_{T_{tra}}^{out}\right\}\in R^{T_{tra}\times d},$$

where $X_{i}^{out}$ denotes the encoded feature of the *i*-th trajectory frame.

#### 3.3.3. Global Trajectory Aggregation and Decoder Reconstruction

To obtain a compact global trajectory representation, masked temporal average pooling is employed:

(8) $$X_{encoded}^{tra}=\frac{1}{T_{tra}}\sum_{i=1}^{T_{tra}}X_{i}^{out}$$

Masked aggregation suppresses interference caused by padded frames while preserving the overall spatial distribution of long-term trajectories. The aggregated trajectory representation is then fed into a transposed-convolution-based decoder, which progressively restores the spatial resolution and generates the reconstructed floorplan $X^{floor}\in R^{3\times H\times W}$.

### 3.4. Motion Feature Extraction Module

The motion feature extraction module aims to extract discriminative spatial representations from short-term radar point cloud sequences. Human actions usually contain diverse motion patterns distributed at multiple spatial scales. For example, falling actions involve obvious large-scale body displacement, whereas sitting or standing states mainly contain slight and subtle posture variations. Therefore, a Feature Pyramid Network (FPN) is introduced to jointly capture fine-grained local motion details and high-level semantic representations.

The overall architecture of the motion feature extraction module consists of hierarchical convolutional encoding, top-down feature fusion, and latent feature projection. Hierarchical convolutional layers with ReLU activation progressively extract multi-level spatial features, denoted by $C_{i}$. The FPN then integrates low-level structural information with high-level semantic features through a top-down pathway:

(9) $$F_{i}=\left\{\begin{array}{cc} \mathrm{Conv}(C_{i}), & i=L,\\ \mathrm{Conv}\left(C_{i}\right)+\mathrm{Up}\left(F_{i+1}\right), & i<L, \end{array}\right.$$

where $C_{i}$ denotes the feature extracted at the *i*-th convolutional level, Up(·) denotes the upsampling operation, and *L* denotes the deepest feature level. As the network depth increases, shallow layers preserve local spatial details, whereas deeper layers capture higher-level semantic motion patterns. Through top-down multi-scale fusion, the network simultaneously models local posture variations and global body movements across different spatial scales.

After multi-scale fusion, the spatial features are flattened and projected into a latent embedding space:

(10) $$X^{act}\in R^{T_{act}\times d}$$

where $T_{act}$ denotes the action sequence length and *d* denotes the embedding dimension. The extracted motion embeddings are subsequently utilized for context-aware action recognition.

### 3.5. Environmental Context Fusion Module

The environmental context fusion module integrates reconstructed scene information with motion features for context-aware human activity recognition.

Actions with similar motion patterns may correspond to different semantic meanings under different environmental contexts. For example, sitting and falling both contain downward body movements, but their surrounding scene structures are substantially different. Therefore, environmental information should be adaptively incorporated into motion representation learning. The scene-dependent structural information is intentionally incorporated as contextual information, since the central motivation of ECRAR is to exploit the relationship between human activities and environmental functional regions. The contextual representation is derived solely from radar-observed trajectory distributions, without using recording identifiers, sequence indices, or action labels as contextual inputs. First, the $X_{encoded}^{tra}$ is used as scene embeddings. Instead of directly concatenating scene features with motion features, a cross-attention mechanism is introduced to dynamically associate human motion with relevant environmental regions.

The encoded trajectory representation $X_{encoded}^{tra}$ is used as the scene embedding for context fusion. The reconstructed floorplan serves as auxiliary supervision for learning environmental representations rather than being converted into discrete region labels for action classification.

Specifically, motion features are treated as queries, while scene features serve as keys and values. The cross-attention mechanism adaptively weights the scene features according to their relevance to the motion representation.

(11) $$Q=X^{act},\;K=V=X_{encoded}^{tra}$$

Compared with direct feature fusion, cross-attention enables the network to adaptively focus on scene regions that are more relevant to current motion patterns, thereby improving human–environment interaction modeling capability. Finally, the output features of cross-attention $X_{cross}^{out}$ are concatenated with the scene embeddings, and the fused representation is formulated as:

(12) $$X^{fusion}=\mathrm{FFN}\left(\mathrm{Concat}\left(X_{cross}^{out},X_{encoded}^{tra}\right)\right)$$

The fused features are subsequently processed by a Transformer encoder and fed into a multilayer perceptron classifier for final action prediction.

### 3.6. Loss Function

#### 3.6.1. Focal Loss

To improve the recognition accuracy for hard samples, Focal Loss is adopted to optimize the ECF and MFE modules. The Focal Loss is computed according to Equation (13).

(13) $$L_{focal}=-\sum_{n=0}^{N-1}\alpha_{n}y_{n}{\left(1-{\hat{y}}_{n}\right)}^{\gamma }\log\left({\hat{y}}_{n}\right)$$

where *N* is the number of action categories, $y_{n}$ is the ground-truth indicator of the *n*-th class, ${\hat{y}}_{n}$ is the corresponding predicted probability, $\alpha_{n}$ is the class-balancing weight, and γ is the focusing parameter.

#### 3.6.2. MMSE Loss

In this work, the floorplan is reconstructed by inverting human motion trajectories. However, since human trajectories cannot fully cover all regions of the scene, directly computing the loss over the entire floorplan would impose supervision on unobserved areas. This may lead to unreasonable predictions, where the model generates scene structures in regions without any trajectory evidence. Therefore, it is necessary to introduce spatial constraints into the loss function. Accordingly, the TFI module is designed to recover trajectory-supported environmental context for assisting HAR, rather than to perform complete geometric reconstruction of the entire indoor environment.

**Figure 3:**
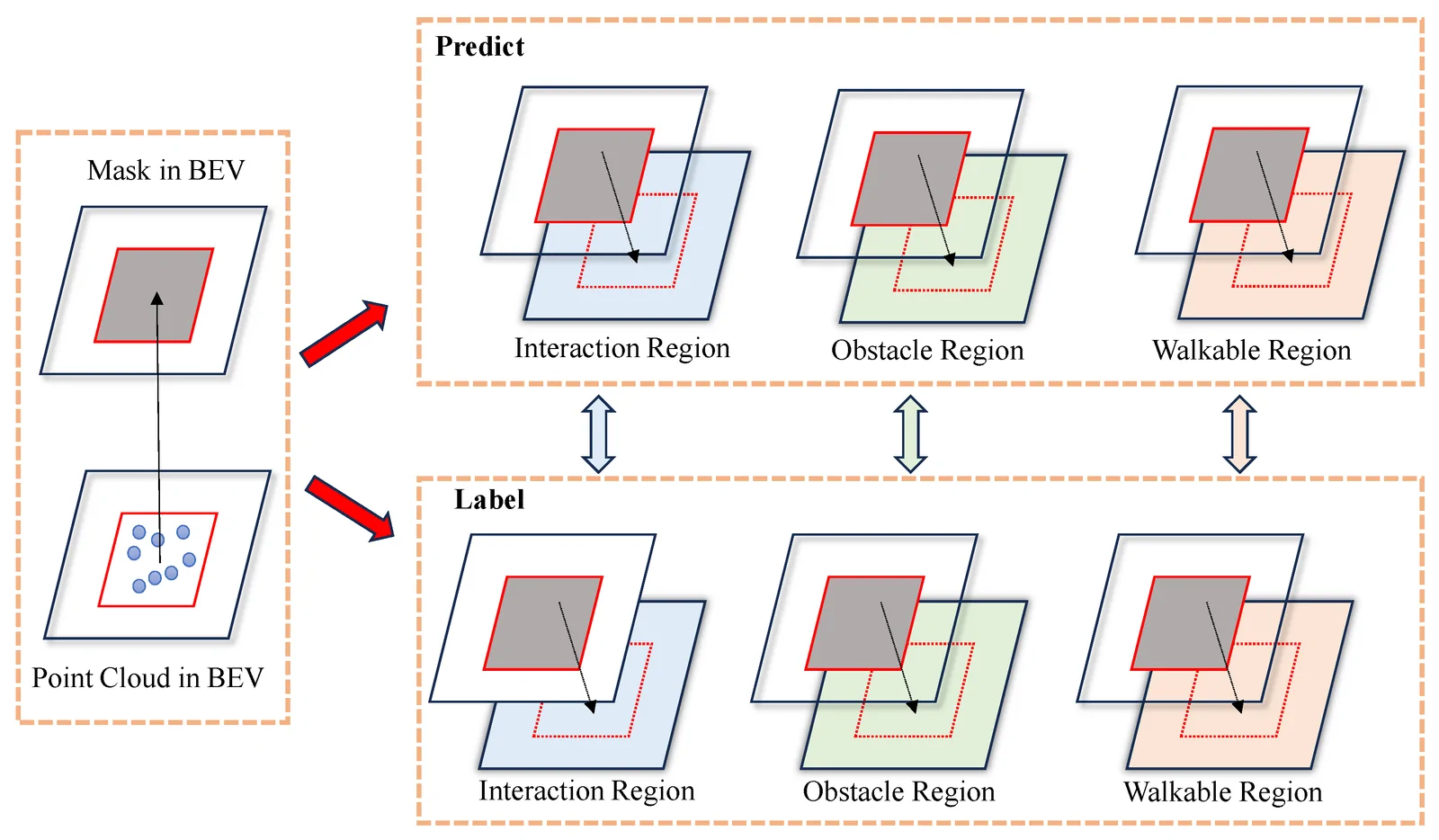
MMSE loss.

We propose the MMSE loss to optimize the TFI module based on point cloud distribution, which computes the loss only in regions where valid trajectory data exist. As shown in Figure 3, a spatial mask *M* is defined for the grid cell at position (i,j):

(14) $$M\left(i,j\right)=\left\{\begin{array}{cc} 1, & D(i,j)>0\\ 0, & D(i,j)=0 \end{array}\right.$$

where D(i,j) denotes the number of trajectory points falling into the (i,j)-th grid cell.

The loss is then computed only over the valid regions indicated by the mask. For each valid grid cell, the mean squared error is calculated across all three channels of the floorplan representation, resulting in the overall loss:

(15) $$L_{\mathrm{MMSE}}=\frac{1}{\sum_{i,j}M\left(i,j\right)}\sum_{c=1}^{3}\sum_{i=1}^{H}\sum_{j=1}^{W}M\left(i,j\right)\cdot {\left(S_{c}\left(i,j\right)-X_{c}^{floor}\left(i,j\right)\right)}^{2}$$

where *c* indexes the three channels of the floorplan representation, $S_{c}\left(i,j\right)$ and $X_{c}^{floor}\left(i,j\right)$ denote the ground-truth and predicted values of channel *c* at grid cell (i,j), respectively, and M(i,j) is the spatial mask defined above.

## 4. InActivity-Scene Dataset

To validate the effectiveness of the proposed context-aware HAR framework, a new mmWave radar dataset, InActivity-Scene, is constructed. Different from existing radar HAR datasets that only provide motion annotations, the proposed dataset simultaneously contains human action samples and explicitly annotated environmental context. Therefore, the dataset not only supports conventional action recognition tasks, but also enables research on scene-aware human activity understanding.

### 4.1. Acquisition Equipment

The InActivity-Scene dataset is collected using an intelligent health monitoring device developed by Beijing Racobit Electronic Information Technology Co., Ltd. (Beijing, China). The device is a commercial 4D mmWave radar sensor based on Frequency Modulated Continuous Wave (FMCW) technology.

Compared with vision sensors, mmWave radar possesses several advantages for indoor HAR, including privacy preservation, robustness under poor illumination conditions, and strong penetration capability. More importantly, 4D mmWave radar can simultaneously provide range, Doppler velocity, azimuth angle, and elevation angle information, enabling the acquisition of fine-grained three-dimensional motion point clouds.

The radar continuously outputs multi-dimensional target information, including: range, Doppler velocity, azimuth angle, elevation angle, and signal-to-noise ratio (SNR). These measurements provide reliable geometric and motion information for subsequent action recognition and environmental reconstruction tasks. The detailed hardware specifications are summarized in Table 1.

**Figure 4:**
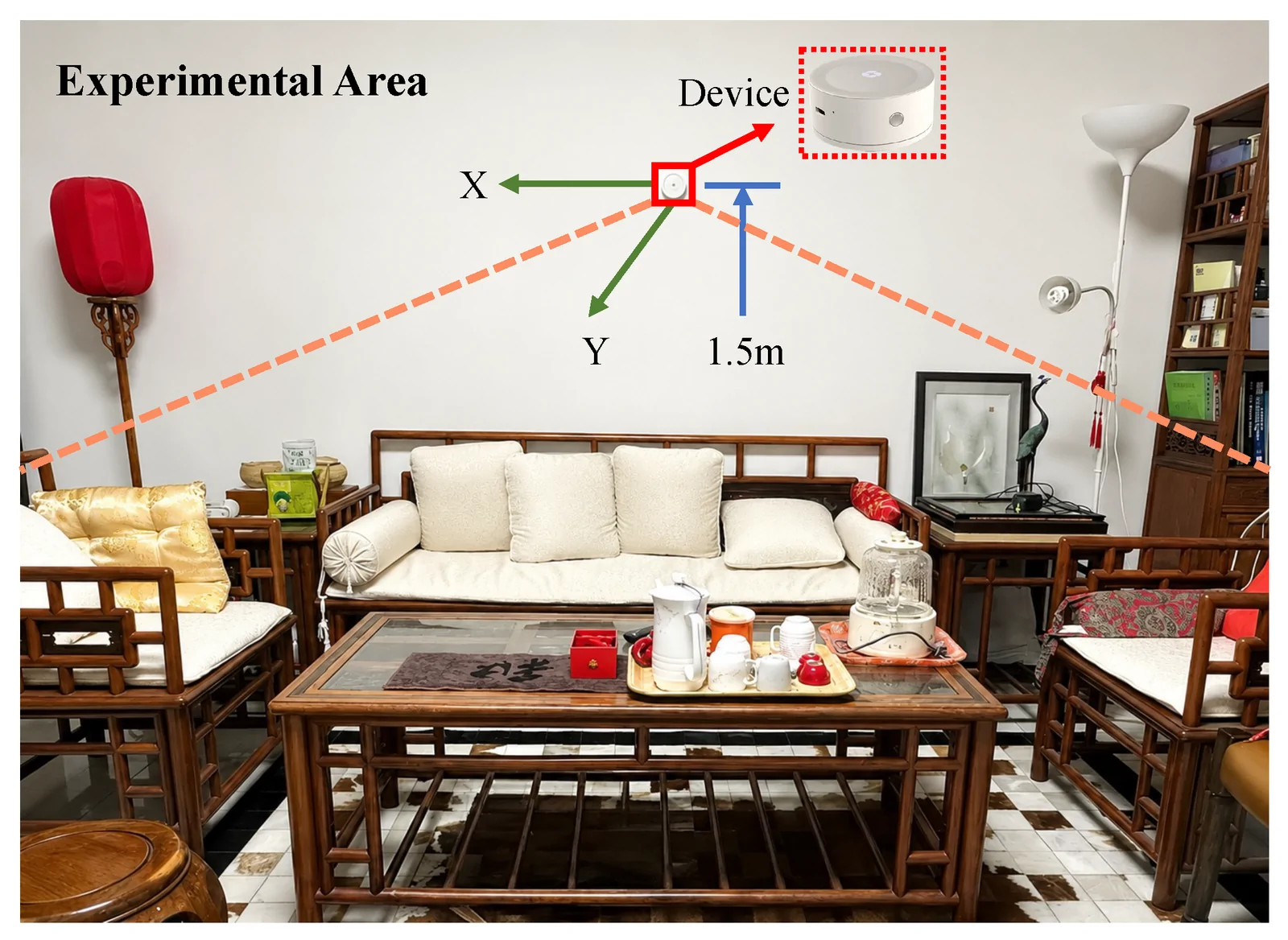
Radar installation configuration.

During actual data collection, the mmWave radar device is mounted on a side wall at a height of 1.5 m above the ground, with its central sensing axis parallel to the ground plane. For each indoor environment, the installation position and sensing orientation are adjusted according to the room layout to ensure that the designated experimental area remains within the effective field of view of the radar. The installation principle is consistently adopted across all scenes, thereby maintaining stable sensing coverage and comparable data acquisition conditions. This configuration ensures effective radar coverage of the designated action-performing area, while reducing the influence of occlusion, angular deviation, and other sensing-related factors on data quality. Although the physical installation position vary across different indoor environments, all radar observations and scene representations are consistently defined in a radar-centered coordinate system, providing a unified spatial reference for subsequent environmental reconstruction and action recognition. A detailed example of the radar installation is shown in Figure 4.

### 4.2. Data Collection Protocol

To improve the diversity and generalization capability of the dataset, data collection is conducted from three aspects, including action diversity, scene diversity, and subject diversity. All experiments are carried out under controlled conditions with standardized action protocols to ensure data consistency and annotation reliability.

#### 4.2.1. Action Categories

**Figure 5:**
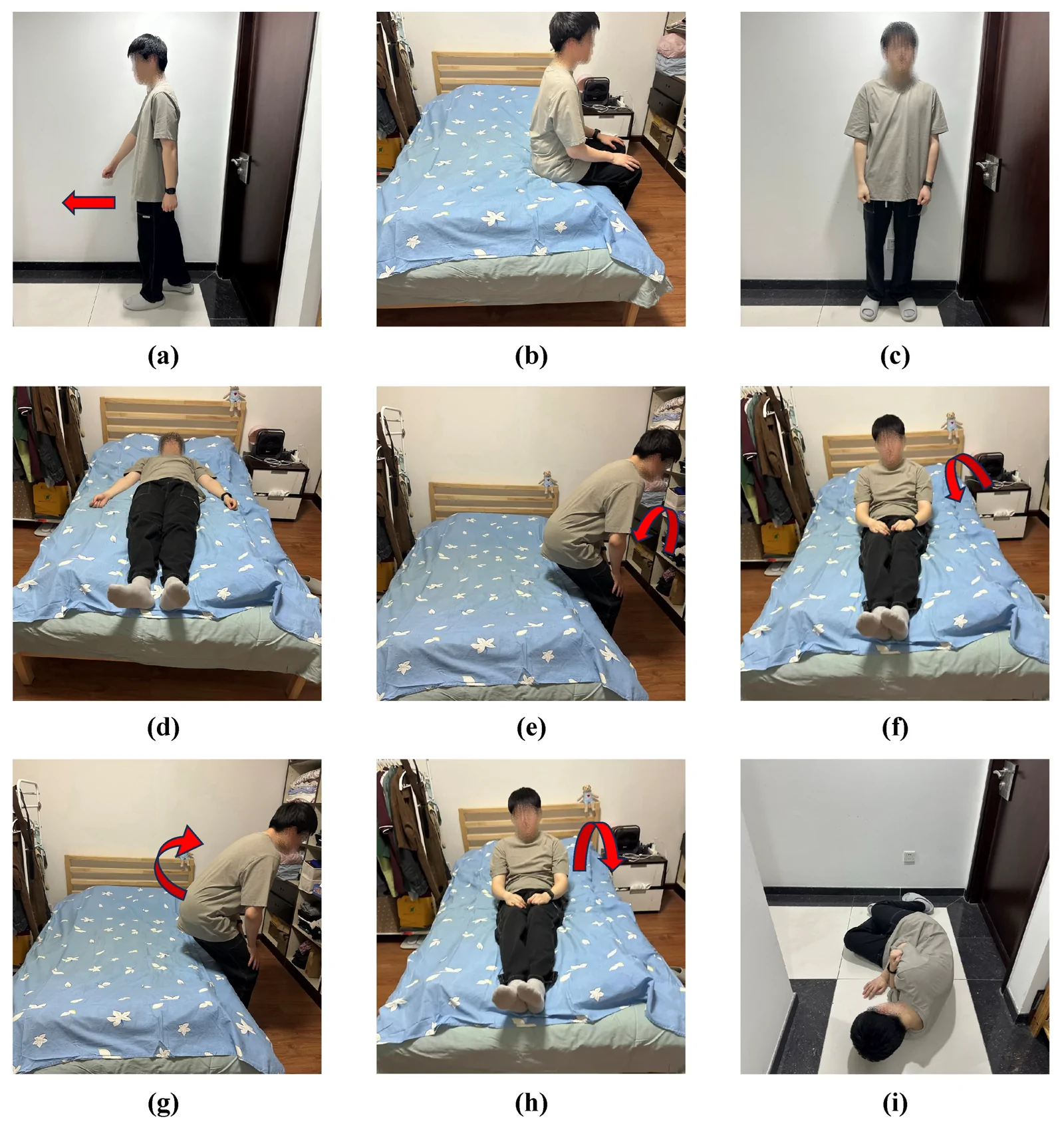
Action categories of InActivity-Scene: (**a**) walk; (**b**) sit; (**c**) stand; (**d**) lie; (**e**) sit down; (**f**) lie down; (**g**) stand up; (**h**) sit up; (**i**) fall.

The dataset contains nine daily human actions, including walk, sit, stand, lie, sit down, lie down, stand up, sit up, and fall. The action categories are illustrated in Figure 5.

The selected actions cover both routine daily behaviors and high-risk emergency events. In particular, falling actions are included because fall detection is one of the most important applications in intelligent healthcare and indoor safety monitoring systems. Moreover, these actions exhibit significant differences in posture transitions, temporal evolution, and motion intensity, providing rich spatiotemporal characteristics for evaluating HAR algorithms.

#### 4.2.2. Scene Diversity

Unlike most existing radar HAR datasets that are collected in limited laboratory environments, the proposed dataset emphasizes environmental diversity. Data are collected from 25 real indoor environments, including 12 bedrooms, 4 living rooms, 4 bathrooms, and 5 kitchens.

These environments exhibit substantial differences in room layouts, spatial scales, furniture distributions, and obstacle configurations. The introduction of scene diversity is important because environmental structure strongly influences human movement patterns and radar reflections. Collecting data across diverse indoor environments improves the robustness and cross-scene generalization capability of the dataset.

#### 4.2.3. Subject Diversity

The dataset involves six participants, including three males and three females, with heights ranging from 163 cm to 180 cm and ages between 25 and 48 years. All participants follow standardized action instructions during data acquisition to ensure consistency and reliability. The standardized acquisition protocol helps reduce unnecessary variations in action execution across participants. In addition, compared with visual observations, sparse 4D mmWave radar point clouds mainly characterize geometric structures and motion patterns rather than appearance-level details, which can reduce the influence of certain individual-specific visual characteristics. As the InActivity-Scene dataset primarily focuses on the relationship between human activities and environmental context, participant diversity is not a primary consideration in the current dataset design.

#### 4.2.4. Data Segmentation Strategy

Different human actions exhibit distinct temporal characteristics. Continuous actions such as walk, sit, stand, and lie mainly involve relatively stable motion states, whereas transient actions such as sit down, stand up, and fall contain abrupt posture transitions and rapid motion changes.

Accordingly, a task-oriented segmentation strategy is adopted. For continuous actions, a non-overlapping sliding window with a window length of 3 s and a stride of 3 s is utilized to preserve complete motion cycles. For transient actions, samples are extracted using a 3-s temporal window centered on the action moment, covering 1 s before and 2 s after the event, thereby retaining both motion transition dynamics and post-action posture characteristics. Samples containing fewer than 5 valid frames are removed to suppress incomplete or noisy data. The final sample distribution is summarized in Table 2. Although the distribution of scene-action combinations varies across indoor environments, the overall sample numbers of the nine action categories remain relatively balanced. The absent combinations mainly reflect the functional constraints of different indoor scenes rather than missing data.

### 4.3. Scene Context Annotation

One major contribution of InActivity-Scene is the introduction of explicit environmental context annotations. Most existing radar HAR datasets only provide action labels while lacking scene structural information. Consequently, they cannot support research on context-aware HAR or human–environment interaction modeling.

To address this limitation, all indoor environments in InActivity-Scene are manually annotated under a Bird’s-Eye View (BEV) coordinate system. The radar position is defined as the coordinate origin, and each scene boundary is represented as:

(16) $$\left[x_{\min},x_{\max},y_{\min},y_{\max}\right]$$

Similarly, all furniture and obstacle regions are annotated as:

(17) $$\left[\mathrm{obj}:x_{\min},x_{\max},y_{\min},y_{\max}\right]$$

In total, 25 indoor environments are annotated, and each scene contains between 1 and 7 objects. The annotated environmental context provides explicit scene constraints for context-aware human activity recognition and trajectory-based floorplan reconstruction.

### 4.4. Dataset Preprocessing

The raw outputs of mmWave radar are represented in spherical coordinates rather than Cartesian coordinates. Therefore, the coordinate transformation is first performed. Given a point *p* with spherical coordinates $\left(r_{p},\theta_{p},\phi_{p}\right)$, where $r_{p}$, $\theta_{p}$, and $\phi_{p}$ denote range, azimuth angle, and elevation angle, respectively, the Cartesian coordinates are computed as:

(18) $$\left\{\begin{array}{cc} x_{p} & =r_{p}\cdot \cos\phi_{p}\cdot \cos\theta_{p}\\ y_{p} & =r_{p}\cdot \cos\phi_{p}\cdot \sin\theta_{p}\\ z_{p} & =r_{p}\cdot \sin\phi_{p} \end{array}\right.$$

To suppress environmental noise and invalid reflections, the valid sensing range is constrained to x∈[−2.5,2.5] m, y∈[0,5] m, and z∈[−1.5,1.5] m. Points outside the valid spatial region are removed.

To maintain consistency between environmental representation and motion modeling, the indoor environment is represented as a structured floorplan:

(19) $$S\in R^{3\times H\times W}$$

Considering the significant correlation between human behaviors and spatial functional zones, we divide the indoor scene into the following three functional regions from the perspective of spatial functional attributes:

1. Walkable Region: The walkable region is an open space where humans can move freely without any objects or obstacles. It has good spatial connectivity and allows basic postures such as walking and standing. Sudden actions such as accidental falls may also occur here, making it the main area for daily human movement.
2. Interaction Region: The interaction region is a functional zone where humans can reach and interact with indoor objects, typically including furniture such as beds, sofas, and chairs. Humans can perform actions with clear interaction intentions (e.g., sitting, lying down, and sitting up) by using these objects. Since behavioral patterns are highly related to object functions, this region serves as the core area for expressing complex daily human activities.
3. Obstacle Region: The obstacle region is a restricted space inaccessible to humans, containing walls, large furniture, or other obstacles. It is physically enclosed and impenetrable. This region does not support human entry or activities; it is used to define scene boundaries and provide spatial constraints.

To convert the continuous scene annotations into the structured grid representation, the BEV plane is uniformly divided into an H×W spatial grid. For each grid cell, the area occupied by each functional region is calculated according to its overlap with the corresponding annotated region. Specifically, for the *c*-th functional region and the grid cell at position (i,j), the area ratio is defined as:

(20) $$S_{c}\left(i,j\right)=\frac{\mathrm{Area}\left(G_{i,j}\cap R_{c}\right)}{\mathrm{Area}\left(G_{i,j}\right)},$$

where $G_{i,j}$ denotes the (i,j)-th grid cell, $R_{c}$ denotes the continuous spatial region corresponding to the *c*-th functional category, and c∈{1,2,3} represents the walkable, interaction, and obstacle regions, respectively. Therefore, each grid cell is represented by three area-ratio values, allowing continuous scene boundaries to be mapped into the discrete grid while preserving sub-grid boundary information.

**Figure 6:**
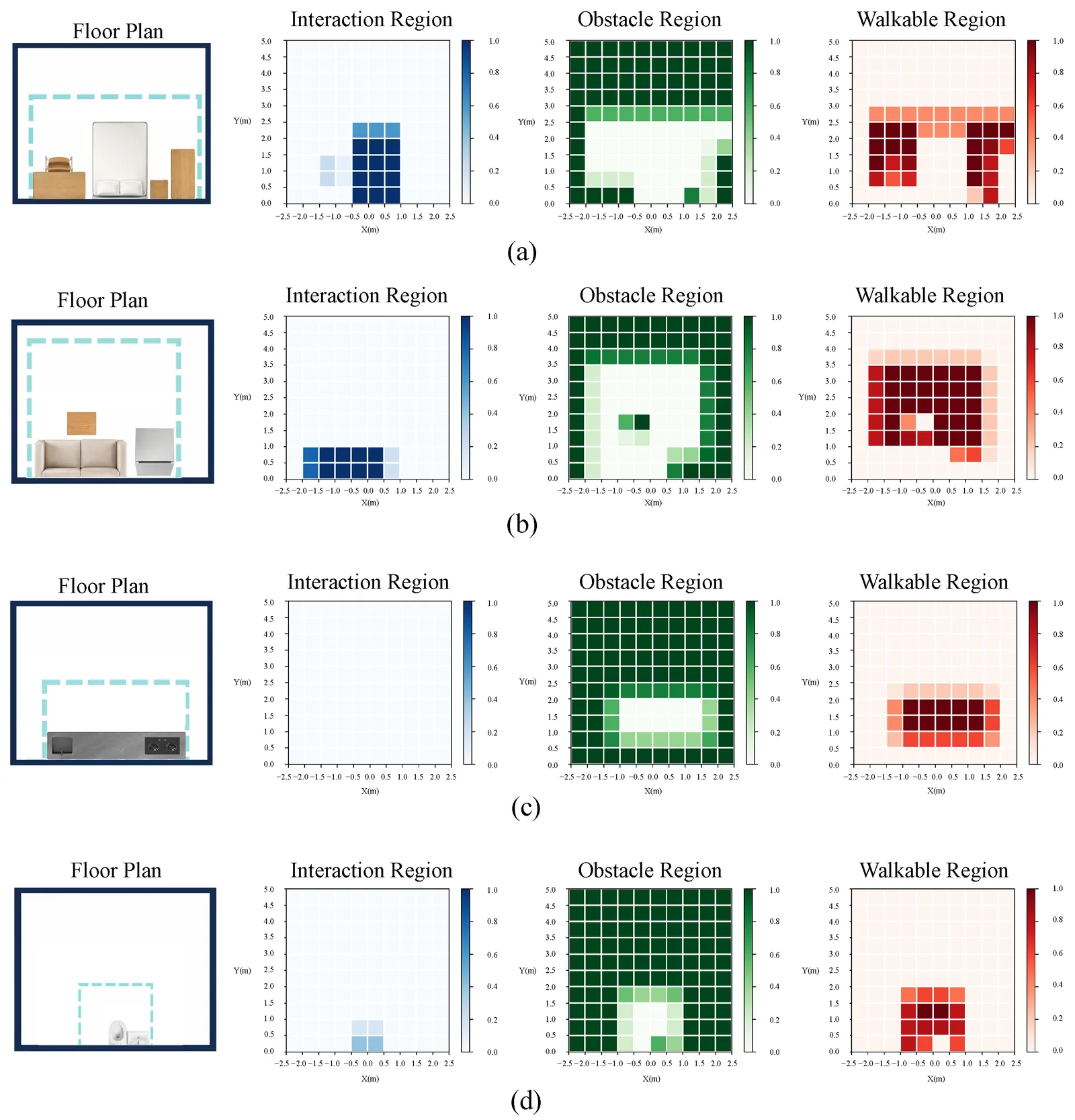
Examples of scene annotations in InActivity-Scene: (**a**) bedroom; (**b**) living room; (**c**) kitchen; (**d**) bathroom.

Compared with assigning each grid cell a single hard categorical label, the proposed three-channel area-ratio representation characterizes the proportional composition of different functional regions within each grid cell, thereby preserving fine-grained boundary information more effectively. Some scenes are illustrated in Figure 6.

## 5. Experiments

### 5.1. Implementation Details

#### 5.1.1. Optimization Details

In the experiment, the overall spatial range is defined as [−2.5m, 2.5m, 0m, 5m, −1.5m, 1.5m], corresponding to $x_{\min},\;x_{\max},\;y_{\min},\;y_{\max},\;z_{\min}$ and $z_{\max}$ in the Cartesian coordinate system with the radar set as the origin. During the pillarization of point cloud data, the entire spatial region is uniformly partitioned into a 10×10 grid, where each grid cell covers an area of 0.5m×0.5m. For each grid cell, point cloud features are characterized by three statistical attributes: the maximum height, the minimum height, and the number of valid points. For human trajectory sequences, the raw point cloud data are aggregated at a 1-s interval, and the length of all trajectory sequences is unified to 60 frames via the padding mask strategy. For human action sequences, random frame dropping is applied to limit the maximum frame rate to 10 frames per second, and all action sequences are standardized to a fixed length of 30 frames using padding masking. Mask vectors are adopted to record the positions of valid frames for both trajectory and action sequences. Accordingly, the input format of point cloud sequences fed into the model is formulated as (seq_len, feature_num, grid_x, grid_y), where seq_len denotes the sequence length, feature_num represents the number of feature channels, and grid_x and grid_y indicate the grid counts along the x-axis and y-axis. Consequently, the input dimension of the human trajectory sequence is (60, 3, 10, 10), while that of the human action sequence is (30, 3, 10, 10).

In the floorplan inversion module, a Feature Pyramid Network is employed to extract spatial features from each frame. After positional encoding is incorporated, a Transformer encoder is utilized to capture temporal dependencies, and transposed convolution is applied to reconstruct the floorplan with a dimension of (3, 10, 10). In the action feature extraction module, the Feature Pyramid Network extracts spatial motion features frame by frame, yielding action features with a size of (30, 128). A cross-attention mechanism is then introduced to fuse environmental context and human motion information, producing fused features of dimension (30, 128). A class token is prepended to the fused sequence, followed by positional encoding. The Transformer encoder further models temporal characteristics and outputs features with the shape (31, 128). Finally, the output feature corresponding to the class token is fed into the classification head to obtain the final recognition result.

For all experiments, the InActivity-Scene dataset is randomly partitioned into training and testing subsets with a ratio of 7:3 at the complete-sequence level. Each action sequence is assigned exclusively to either the training or the test subset, ensuring that no temporal segment from a test sequence is included in the training data. The same partition is kept fixed throughout all comparative and ablation experiments to ensure a fair and consistent performance comparison. All model training is conducted on a single NVIDIA GeForce RTX 3090 GPU (NVIDIA Corporation, Santa Clara, CA, USA). The batch size is set to 16, and the total number of training epochs is 100. The AdamW optimizer is adopted for parameter optimization, with an initial learning rate of $10^{-4}$ and a weight decay of $10^{-6}$. Dropout regularization is applied to mitigate overfitting, with the dropout rate set to 0.1. During inference, each test sample is processed independently, and its environmental context is constructed from the corresponding radar trajectory observations without accessing other test samples, ground-truth action labels, or classification results.

#### 5.1.2. Evaluation Metrics

In this experiment, Accuracy, Macro-Precision, Macro-Recall, and Macro-F1 are adopted as the model evaluation metrics. When calculating the metric values for the *i*-th category, samples of the *i*-th class are regarded as positive samples, while all other categories are treated as negative samples. The Precision, Recall, and F1-score of the *i*-th class, denoted as ${\mathrm{Precision}}_{i}$, ${\mathrm{Recall}}_{i}$, and ${F1\text{-}\mathrm{score}}_{i}$, are computed according to Equation (21), Equation (22), and Equation (23), respectively. Assuming there are *N* categories in total, the Macro-Precision, Macro-Recall, and Macro-F1 score can be derived from Equation (24) to Equation (26).

(21) $$\mathrm{Precision}=\frac{\mathrm{TP}}{\mathrm{TP}+\mathrm{FP}}$$

(22) $$\mathrm{Recall}=\frac{\mathrm{TP}}{\mathrm{TP}+\mathrm{FN}}$$

(23) $$F1\text{-}\mathrm{score}=2\times \frac{\mathrm{Precision}\times \mathrm{Recall}}{\mathrm{Precision}+\mathrm{Recall}}$$

(24) $$\mathrm{Macro}\text{-}\mathrm{Precision}=\frac{1}{N}\sum_{i=1}^{N}{\mathrm{Precision}}_{i}$$

(25) $$\mathrm{Macro}\text{-}\mathrm{Recall}=\frac{1}{N}\sum_{i=1}^{N}{\mathrm{Recall}}_{i}$$

(26) $$\mathrm{Macro}\text{-}F1=\frac{1}{N}\sum_{i=1}^{N}{F1\mathrm{Score}}_{i}$$

where TP, FP, and FN denote the numbers of true positives, false positives, and false negatives, respectively.

### 5.2. Performance Evaluation

#### 5.2.1. Overall Performance

**Figure 7:**
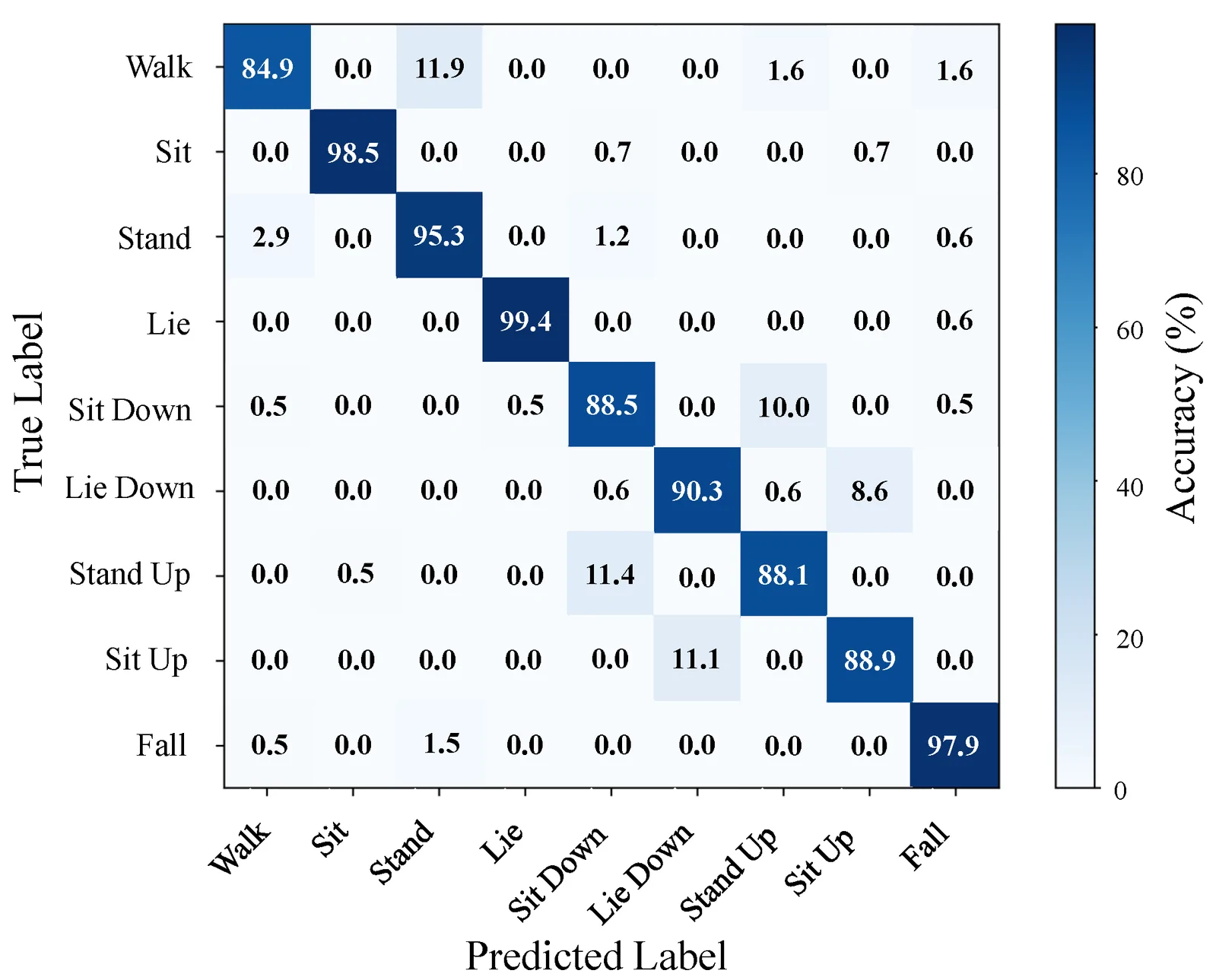
Confusion matrix for ECRAR on the InActivity-Scene dataset.

On the InActivity-Scene dataset, the recognition accuracy of the proposed method reaches 92.2%, with a macro-averaged precision of 92.5%, a macro-averaged recall of 92.4%, and a macro-averaged F1-score of 92.4%. The confusion matrix of the recognition results obtained by ECRAR is illustrated in Figure 7. It can be observed that the model achieves high recognition accuracy for the actions of lie, sit, and fall.

#### 5.2.2. Comparative Results

To validate the effectiveness of the proposed method, extensive experiments are conducted on the InActivity-Scene dataset. We compare ECRAR with several representative HAR methods, including SVM, MLP, BiLSTM, and TDCNN_BiLSTM [39], as well as m-Activity [30]. In addition, ECRAR (w/o E), which excludes environmental context information while retaining the motion-based recognition framework, is introduced to specifically evaluate the contribution of environmental context. All methods are evaluated using the same fixed sequence-level data partition and experimental settings to ensure a consistent performance comparison. The quantitative comparison results of human action recognition are presented in Table 3. It can be observed that the proposed ECRAR method achieves the best performance across all evaluation metrics, which verifies the superiority and effectiveness of the proposed approach.

In particular, under the same data partition and training conditions, ECRAR improves the recognition accuracy from 83.8% for ECRAR (w/o E) to 92.2%, demonstrating that the proposed environmental context modeling provides complementary discriminative information beyond motion features alone. Moreover, ECRAR outperforms all other baseline methods in terms of overall recognition accuracy. Specifically, SVM and MLP lack temporal modeling mechanisms and rely merely on isolated spatial features for action recognition. Consequently, they fail to capture the dynamic temporal dependencies of human movements and cannot attain satisfactory recognition accuracy. The BiLSTM model directly performs temporal modeling on 3D voxel features without a dedicated spatial feature extraction module, making it insufficient to fully exploit spatial motion characteristics and resulting in limited recognition performance. Although TDCNN_BiLSTM and m-Activity both integrate spatial and temporal modeling modules, their inherent deficiencies in spatial and temporal feature extraction restrict the comprehensive mining of spatiotemporal cues, thereby yielding slightly lower overall accuracy than our method.

#### 5.2.3. Performance Analysis Across Diverse Scenarios

To further investigate the influence of environmental context on radar-based human action recognition, we systematically compare recognition performance with and without environmental context fusion. In-depth analyses are further conducted at the fine-grained action category level. Table 4 summarizes the per-class recognition accuracy of the baseline ECRAR (w/o E) model and the proposed scene-aware ECRAR model.

Experimental results demonstrate that, compared with the method without environmental context, the model embedded with environmental semantic information achieves accuracy gains across nearly all action categories except walking. The slight decrease in walking accuracy may be attributed to its weak dependence on specific environmental contexts, as walking can occur across different functional regions and is primarily characterized by continuous motion patterns. Therefore, additional scene information provides limited complementary cues and may introduce slight contextual interference. In contrast, the performance improvement is particularly prominent for environment-interactive and context-dependent actions such as sitting down, lying down, and sitting up. This phenomenon indicates that environmental context provides effective spatial constraints and interaction cues for actions that are closely associated with specific indoor regions or objects. By incorporating such contextual information, the model enhances its discriminative capability for environment-dependent actions, thereby improving the overall performance.

**Figure 8:**
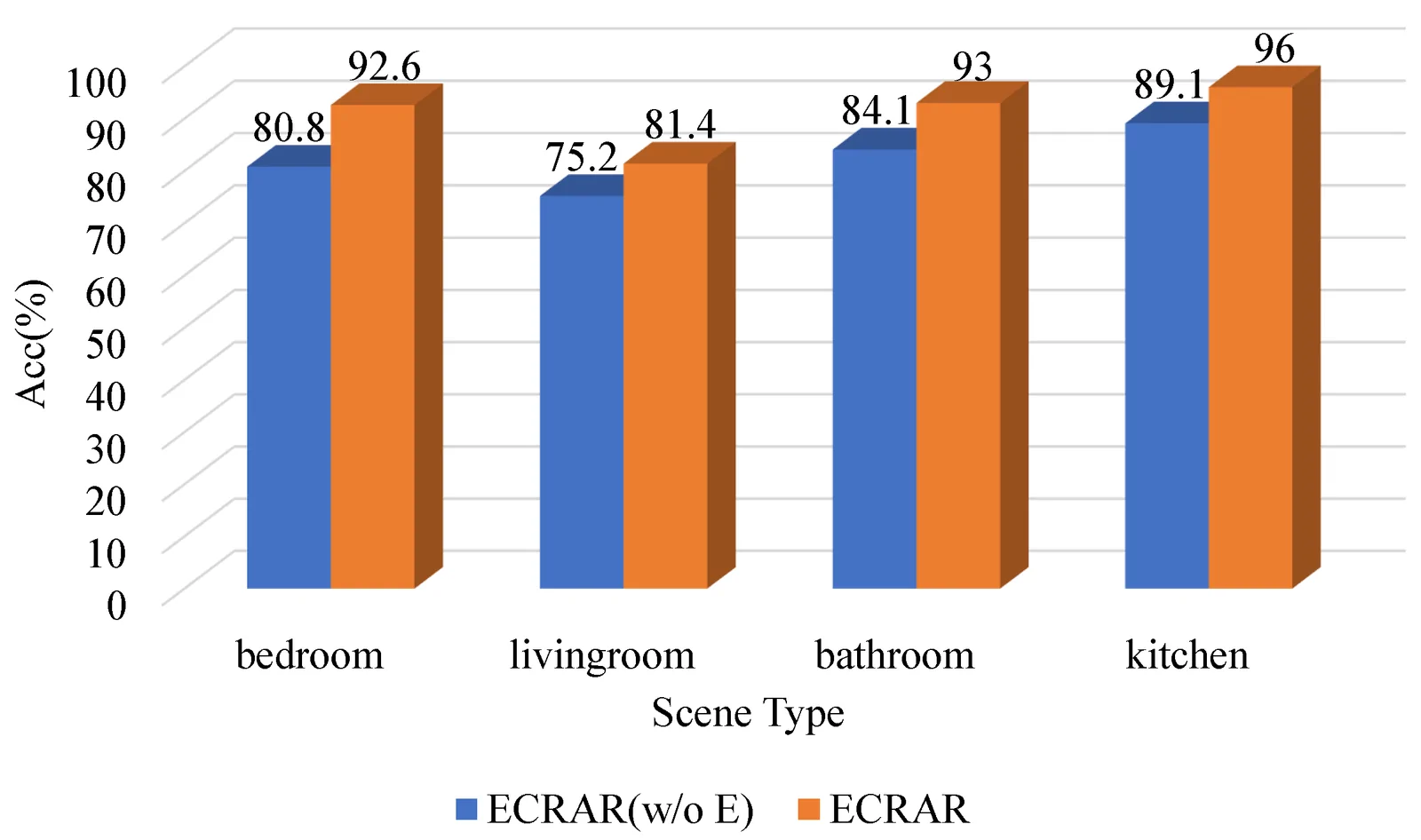
Action recognition accuracy statistics for different scenes.

To explore the intrinsic correlation between recognition accuracy and environmental complexity, performance comparisons are conducted across four typical indoor scenarios: bedroom, living room, kitchen, and bathroom. The corresponding experimental results are presented in Figure 8. It is evident that incorporating environmental context consistently improves recognition accuracy in all scene types, with the most remarkable gain of 11.8% observed in bedroom scenarios. Moderate accuracy improvements are also achieved in living room, bathroom, and kitchen environments.

In detail, bedrooms generally feature larger spatial ranges, more intricate layouts, and more frequent human–furniture interactions, accompanied by richer and more diverse action patterns. Such complex scenarios pose considerable challenges to recognition methods that solely rely on human motion point clouds. The experimental results validate that by fusing environmental context information, the model can effectively perceive scene structural layouts and exploit environmental constraints. This allows the model to better capture the inherent correspondence between human actions and scene layouts, and substantially enhances action discrimination ability and recognition accuracy in complex indoor environments.

**Figure 9:**
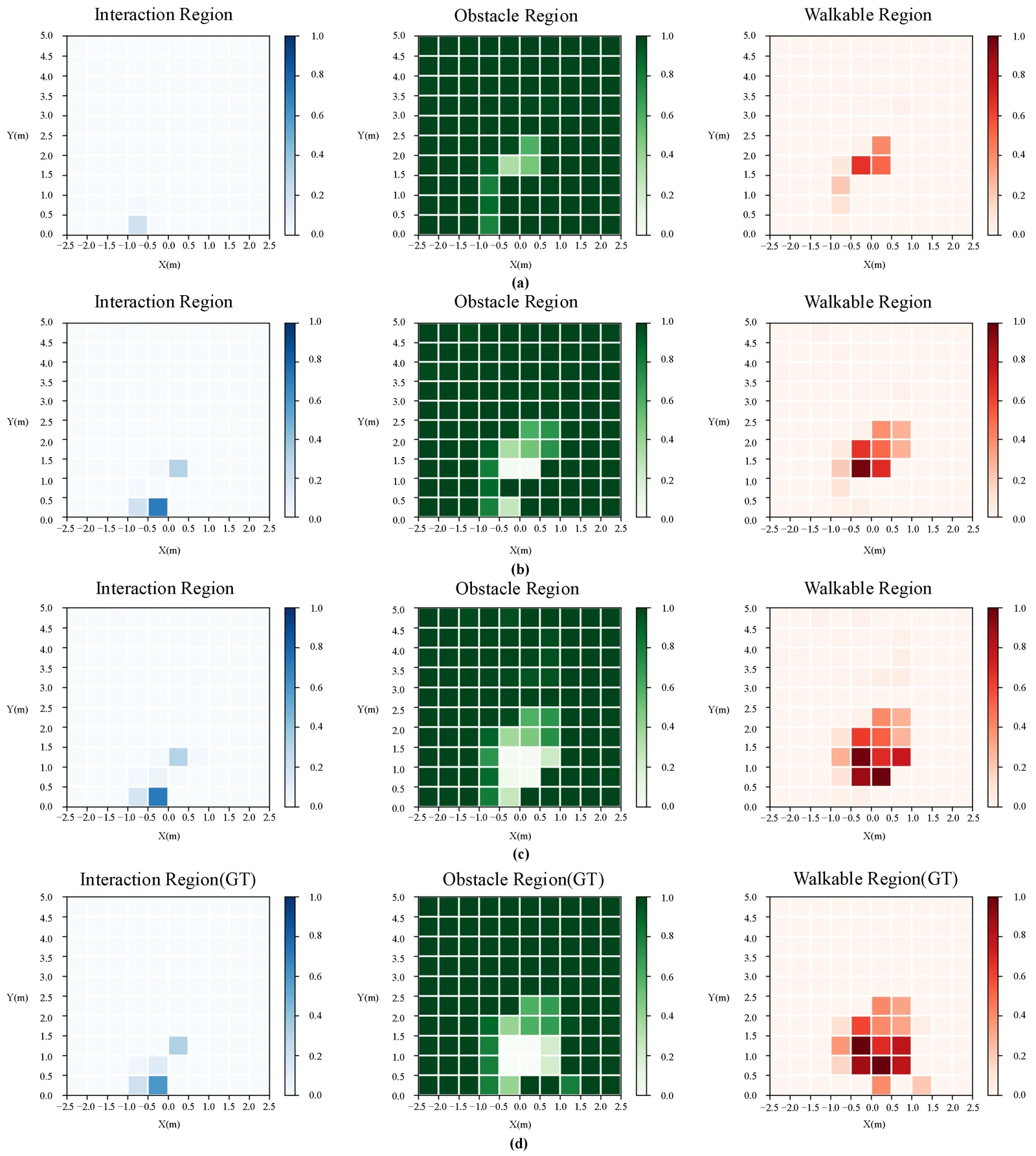
Dynamic update of environmental context: (**a**) 30 s; (**b**) 60 s; (**c**) 120 s; (**d**) Ground Truth.

The proposed method enables dynamic updating of the environmental context. At the model initialization stage, the overall area is defaulted to a full obstacle layout as the initial scene state. With the continuous input of mmWave radar point cloud streams, the model can progressively update the functional information of regions covered by point clouds. To intuitively demonstrate the performance of the proposed method in floorplan inversion, Figure 9 visualizes the dynamic evolution of environmental context from the initial state to different time steps. As additional trajectory observations provide broader spatial coverage, the reconstructed floorplan is progressively supplemented and refined, exhibiting increasingly complete spatial structures, while the annotated ground truth is provided for qualitative comparison. This visualization illustrates the feasibility of trajectory-based environmental reconstruction. Since the environmental context is reconstructed from accumulated trajectory observations rather than relying on a fixed pre-stored map, this update mechanism also allows the scene representation to adapt to layout changes as new spatial evidence becomes available.

### 5.3. Ablation Study

To verify the effectiveness of the proposed method, ablation experiments on the ECRAR framework are performed in this section, covering six comparative dimensions: hyperparameters, spatial feature extractor, temporal feature extractor, human trajectory duration, floorplan representation, and cross-attention mechanism.

#### 5.3.1. Impact of Hyperparameters

To evaluate the influence of hyperparameter settings on recognition performance, ablation experiments are conducted on the learning rate (lr) and the feature mapping dimension (dim) of the spatial feature extraction module. The experimental results are summarized in Table 5. Different hyperparameter configurations lead to noticeable variations in model performance. When the learning rate is excessively large, the training process becomes unstable and may converge to suboptimal solutions, while overly small learning rates slow down convergence and limit representation learning capability. In addition, the mapping dimension directly affects the feature representation capacity of the network. Smaller dimensions may lead to insufficient semantic representation, whereas excessively large dimensions introduce redundant features and increase computational complexity. Among all configurations, the proposed model achieves the best recognition performance when the learning rate is set to $1\times 10^{-4}$ and the mapping dimension is set to 128, which validates the rationality of the selected hyperparameter settings. Moreover, under reasonable learning-rate settings of $5\times 10^{-4}$ and $1\times 10^{-4}$, the model maintains recognition accuracies ranging from 90.1% to 92.2% across feature dimensions of 64, 128, and 256. These results indicate that the overall performance is relatively stable across different reasonable hyperparameter configurations rather than being dependent on a single specific setting.

#### 5.3.2. Impact of Spatial Feature Extractor

To verify the contribution of the spatial feature extraction module to human action recognition performance, an ablation model based on a traditional two-dimensional convolutional neural network is constructed for comparative experiments. The experimental results are presented in Table 6. It can be seen that replacing the FPN with an ordinary 2D CNN reduces the overall accuracy by 1.8%. This demonstrates that the spatial feature extraction module can effectively capture the spatial distribution and posture structural information of human actions contained in radar point clouds, and plays a critical role in improving model recognition accuracy. By fusing multi-scale features, FPN is able to mine spatial information more comprehensively, thereby providing more expressive feature representations for human action recognition.

#### 5.3.3. Impact of Temporal Feature Extractor

To validate the effectiveness of the temporal feature extraction module for human action recognition, ablation experiments are conducted by replacing the Transformer encoder with GRU and LSTM networks, respectively. The experimental results are listed in Table 7. Compared with the proposed model, the recognition accuracy decreases by 2.8% and 2.2% when adopting GRU and LSTM, respectively, while metrics including precision, recall, and F1-score also decline to varying degrees. The experimental results indicate that recurrent neural networks exhibit limited capability in modeling long-range temporal dependencies. In contrast, our temporal feature extraction module more effectively captures temporal dependencies in human actions, enhancing sequence modeling capability.

#### 5.3.4. Impact of Human Trajectory Duration

The ECRAR method adopts 1-min human trajectory data to implement environmental context inversion. To explore how trajectory duration affects context reconstruction and action recognition performance, comparative experiments are conducted using trajectory sequences of 2 min, 30 s, and 15 s. The corresponding recognition results are presented in Table 8. The experimental results show that when the trajectory duration is reduced to 15 s and 30 s, the overall recognition accuracy declines noticeably, accompanied by degradation in macro-averaged precision, macro-averaged recall, and macro-averaged F1-score. By contrast, extending the trajectory duration to 2 min yields no significant performance difference compared with the 1-min setting.

**Figure 10:**
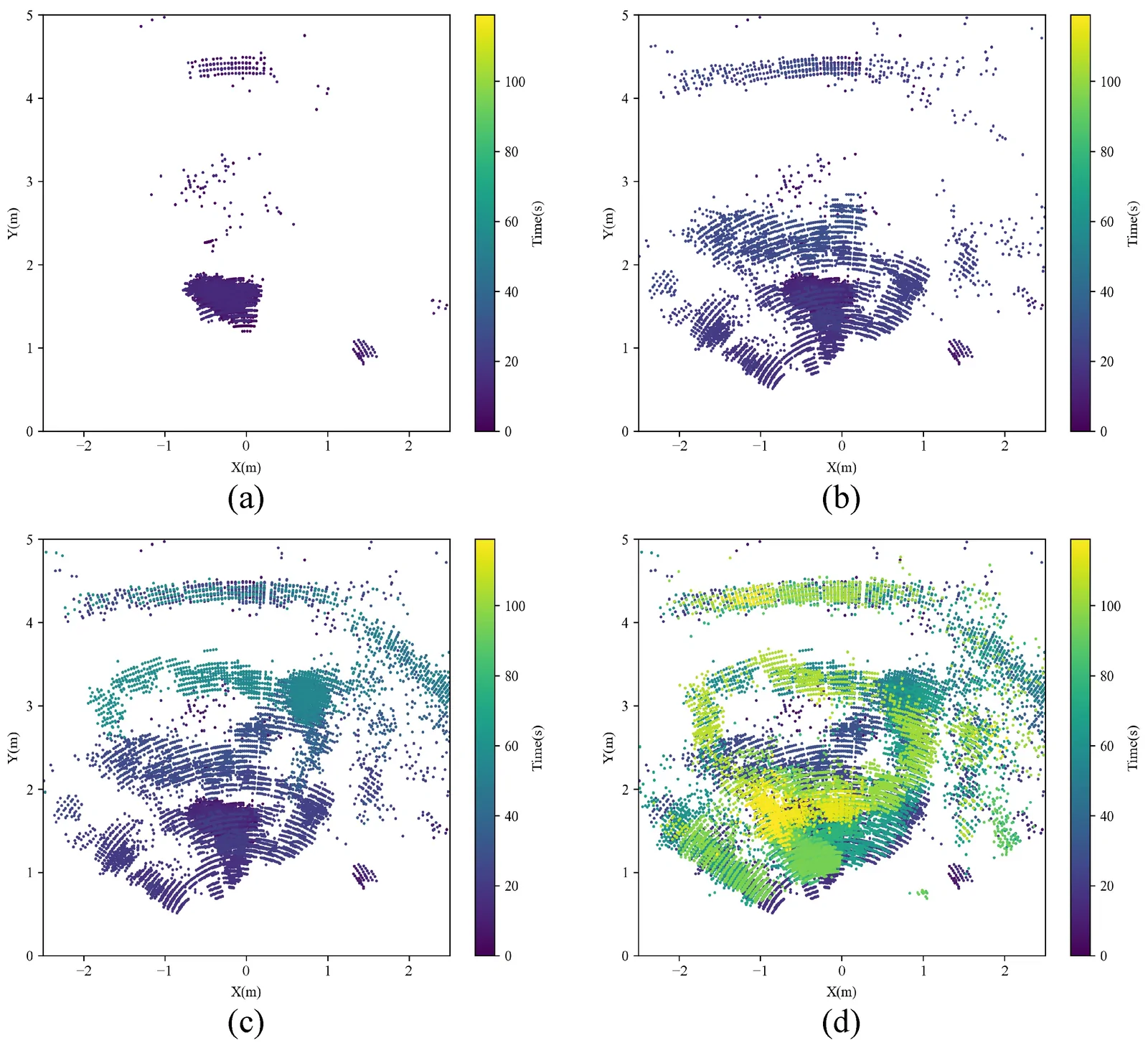
Scatter plots of human trajectories with different durations: (**a**) 15 s; (**b**) 30 s; (**c**) 60 s; (**d**) 120 s.

Figure 10 illustrates the point cloud distribution and spatial coverage obtained under different trajectory durations. The results indicate that the environmental information available for floorplan inversion is fundamentally determined by the spatial coverage of human trajectories rather than the temporal duration alone. In the collected sequences, extending the observation window from 15 s to 60 s provides more opportunities for subjects to traverse different functional regions, resulting in progressively broader spatial coverage. However, further extending the duration to 120 s produces little additional coverage, and the newly observed effective spatial information remains limited.

From the perspective of the proposed HAR framework, complete coverage of the entire indoor environment is not required. The reconstructed environmental context is mainly used to provide spatial constraints and interaction cues relevant to the current action, while the cross-attention mechanism adaptively emphasizes scene features associated with the motion representation. Therefore, as long as the long-term trajectory provides sufficient coverage of action-relevant functional regions, the inferred environmental information can effectively support action recognition. Experimental results further show that the model remains effective under reduced environmental spatial coverage. Even with a 15 s trajectory, where the observed environmental region is substantially more limited, ECRAR achieves an accuracy of 87.2%, which is still higher than the 83.8% accuracy of ECRAR (w/o E). As the trajectory duration increases, broader spatial coverage provides more complete environmental context and further improves recognition performance, with the 1-min trajectory achieving 92.2% accuracy. Extending the observation duration beyond 1 min introduces little additional valid coverage and yields no further performance improvement, while incurring extra computational overhead. Considering both recognition performance and computational efficiency, a 1-min trajectory is adopted in the current implementation.

#### 5.3.5. Impact of Floorplan Representation

To investigate the influence of different floorplan encoding schemes on model performance, the original three-channel area-ratio floorplan is replaced with a single-channel categorical floorplan. In this alternative scheme, each grid cell is assigned only the dominant functional category that occupies the largest area proportion within the cell. The ablation results are listed in Table 9.

It is evident that switching to the single-channel categorical representation leads to a substantial drop across all four evaluation metrics. Constrained by the complexity of real indoor layouts and fixed grid granularity, the boundaries of functional regions rarely align perfectly with regular grid partitions. Consequently, the single-channel representation loses fine-grained details of mixed functional regions within individual grid cells. In comparison, the three-channel area-ratio floorplan characterizes the proportional composition of different functional regions within each grid cell, thereby preserving fine-grained environmental information. The substantial performance difference between the single-channel and three-channel representations further demonstrates that continuous and fine-grained environmental representation provides more effective contextual cues for HAR than coarse categorical region assignment.

#### 5.3.6. Impact of Cross-Attention Mechanism

To verify the contribution of the cross-attention layer in the environmental context fusion module to feature aggregation and recognition performance, an ablation variant is constructed by removing the cross-attention mechanism. Instead, environmental context features and human motion features are fused through direct feature concatenation. The comparative results shown in Table 10 indicate that removing cross-attention degrades the model’s recognition accuracy, which confirms that the cross-attention mechanism effectively enhances feature fusion capability and final recognition performance. Compared with naive feature concatenation, cross-attention enables adaptive dynamic weighting and correlation modeling between motion and environmental context features. It automatically focuses on environment regions and key features highly relevant to ongoing actions, thereby strengthening the quality of multimodal feature fusion.

## 6. Discussion

The experimental results demonstrate that the proposed ECRAR framework effectively improves 4D mmWave radar-based human action recognition by jointly modeling human motion dynamics and indoor scene context. By introducing environmental context through trajectory-to-floorplan inversion and adaptively fusing it with motion features, ECRAR achieves an overall accuracy of 92.2%, clearly outperforming both conventional baselines and the context-free variant ECRAR (w/o E) (83.8%). This confirms our central hypothesis that indoor human activities are strongly coupled with surrounding scene structures, and that explicitly modeling this coupling provides discriminative cues beyond motion dynamics alone.

A closer analysis reveals that the performance gains are concentrated on environment-dependent and interactive actions. Compared with the context-free baseline, accuracy improves most notably for lie (+10.3%), sit down (+14.0%), lie down (+12.6%), and fall (+12.3%), whereas walking, which is largely independent of specific scene structures, shows a marginal decrease. This pattern indicates that the environmental context primarily resolves ambiguities among actions that share similar motion patterns but occur in distinct functional regions, which is precisely the limitation of motion-only methods identified in the Introduction. The scene-level analysis further supports this finding: incorporating environmental context consistently improves accuracy across all four indoor scenarios, with the largest gain of 11.8% observed in bedrooms, where layouts are more complex and human–furniture interactions are more frequent.

The ablation studies provide additional insight into the contribution of each component. The three-channel area-ratio floorplan substantially outperforms both the single-channel categorical representation and the no-floorplan baseline, showing that fine-grained quantification of functional regions is essential for effective context modeling. The cross-attention fusion surpasses naive feature addition and concatenation, indicating that adaptive correlation modeling between motion and scene features is more effective than static fusion. Moreover, the trajectory analysis shows that environmental reconstruction is primarily determined by trajectory spatial coverage rather than temporal duration alone. Under the movement patterns observed in our dataset, a one-minute observation window provides sufficient effective coverage, while longer observations offer marginal performance gains at additional computational cost, resulting in a favorable trade-off between recognition accuracy and computational efficiency. Together, these results validate the effectiveness of sensing indoor scene structures directly from radar point clouds, without any additional sensing devices.

Despite these advantages, several limitations remain. From the data perspective, the current dataset contains only six participants, which limits a comprehensive evaluation of inter-individual generalization. Although the present study primarily focuses on scene diversity and environmental context across different indoor environments, a larger participant population is still necessary to more rigorously evaluate robustness to individual variability. In addition, the InActivity-Scene dataset is collected exclusively under single-person scenarios, which differs from the multi-occupant environments encountered in practical applications such as nursing homes, and the action categories are annotated at a relatively coarse granularity. From the algorithmic perspective, the floorplan representation is sensitive to grid granularity: an overly fine grid may cause spatial misalignment between the reconstructed floorplan and the raw radar point clouds due to the inherent systematic error of mmWave radar, whereas an overly coarse grid may mix multiple functional regions within a single cell and reduce the precision of context representation.

Despite these advantages, several limitations remain. From the data perspective, the current dataset contains only six participants, which limits a comprehensive evaluation of inter-individual generalization. Although the present study primarily focuses on scene diversity and environmental context across different indoor environments, a larger participant population is still necessary to more rigorously evaluate robustness to individual variability. In addition, the InActivity-Scene dataset is collected exclusively under single-person scenarios, which differs from the multi-occupant environments encountered in practical applications such as nursing homes, and the action categories are annotated at a relatively coarse granularity. From the deployment perspective, the current evaluation is conducted under a standardized radar mounting height of 1.5 m, and robustness to mounting-height variations has not yet been systematically evaluated. Moreover, although the preprocessing and temporal aggregation strategies provide basic noise suppression, robustness under different noise conditions has not been quantitatively investigated.

Future work will therefore proceed along two directions. First, the participant population will be expanded to cover broader variations in body characteristics, motion habits, and action patterns, thereby further enriching the diversity of the dataset. Meanwhile, a multi-person point cloud segmentation module based on clustering and feature differentiation will be introduced to separate individual targets and extend ECRAR to multi-occupant scenarios, together with the collection of finer-grained action samples to enrich behavioral diversity. Second, more robust environmental modeling and feature fusion strategies will be explored to further improve recognition performance. The framework will also be extended to accommodate more challenging deployment conditions, including mounting-height variations, stronger radar noise, and dynamic changes in indoor layouts. For applications requiring complete floorplan reconstruction as an independent output, dedicated reconstruction architectures, objectives, evaluation metrics, and comparisons with alternative reconstruction methods can be explored in future work.

## 7. Conclusions

In this paper, we propose ECRAR, an environmental context-aware framework for indoor scene sensing and human action recognition from 4D mmWave radar point clouds. ECRAR adopts a dual-branch design: a trajectory-to-floorplan inversion branch reconstructs indoor scene layouts by modeling the spatiotemporal characteristics of long-term human trajectory point clouds, while a motion feature extraction branch captures discriminative short-term motion representations through multi-scale spatial modeling. A cross-attention-based environmental context fusion module then adaptively integrates the reconstructed scene features with motion features, enabling context-aware action recognition that explicitly exploits human–environment interactions.

To support this study, we construct the InActivity-Scene dataset, in which indoor scenes and internal objects are finely annotated, and environmental context is structured using a three-channel area-ratio floorplan representation. Extensive comparative experiments and ablation studies demonstrate that ECRAR consistently outperforms existing baseline methods and confirm the complementary role of environmental context in mmWave radar-based human action recognition, particularly for environment-dependent actions such as sitting down, lying down, and falling. These results indicate that the proposed framework and dataset provide a useful foundation for future research on privacy-preserving indoor radar sensing and scene-aware human activity understanding.

## Figures and Tables

**Table 1:** Key parameters of the 4D mmWave radar used for data acquisition.

Parameter	Value
Center frequency	61 GHz
Bandwidth	2 GHz
Range resolution	∼0.08 m (with windowing)
Sensing angle	≤120°
Sensing distance	≤7 m
Sensing range	≤40 m^2^
Communication mode	Wi-Fi
Data rate	10 fps

**Table 2:** Statistics of action samples in InActivity-Scene.

Scene Type	Walk	Sit	Stand	Lie	Sit Down	Lie Down	Stand Up	Sit Up	Fall
bedroom	620	869	652	953	745	877	736	872	628
livingroom	224	264	232	249	251	228	248	225	210
bathroom	179	127	190	0	210	0	208	0	184
kitchen	230	0	235	0	0	0	0	0	226
total	1253	1260	1309	1202	1206	1105	1192	1097	1248

**Table 3:** Comparison of HAR accuracy across different methods.

Method	Accuracy	Macro-Precision	Macro-Recall	Macro-F1
SVM	67.7%	70.7%	68.2%	68.1%
MLP	68.0%	69.6%	68.5%	67.7%
BiLSTM	80.6%	80.8%	80.7%	80.6%
TDCNN_BiLSTM	81.3%	81.9%	81.6%	81.6%
m-Activity	82.9%	83.5%	82.9%	83.0%
ECRAR (w/o E)	83.8%	83.9%	84.2%	83.9%
ECRAR (Ours)	**92.2%**	**92.5%**	**92.4%**	**92.4%**

*Note:* The best results are shown in bold, and the second-best results are underlined.

**Table 4:** Recognition accuracy of various actions before and after fusing environmental context.

Action	ECRAR (w/o E)	ECRAR	Accuracy Change
Walk	86.5%	84.9%	−1.6%
Sit	96.3%	98.5%	+2.2%
Stand	90.1%	95.3%	+5.2%
Lie	89.1%	99.4%	+10.3%
Sit Down	74.5%	88.5%	+14.0%
Lie Down	77.7%	90.3%	+12.6%
Stand Up	80.0%	88.1%	+8.1%
Sit Up	77.8%	88.9%	+11.1%
Fall	85.6%	97.9%	+12.3%

**Table 5:** Ablation experiment of hyperparameters.

lr	dim	Accuracy	Macro-Precision	Macro-Recall	Macro-F1
$5\times 10^{-4}$	64	91.7%	92.1%	92.0%	91.9%
$10^{-4}$	64	90.1%	90.5%	90.3%	90.4%
$10^{-5}$	64	78.0%	79.2%	78.8%	78.7%
$5\times 10^{-4}$	128	91.4%	92.0%	91.7%	91.7%
$10^{-4}$	128	**92.2%**	**92.5%**	**92.4%**	**92.4%**
$10^{-5}$	128	86.7%	87.4%	87.1%	87.2%
$5\times 10^{-4}$	256	91.8%	92.1%	91.9%	92.0%
$10^{-4}$	256	91.0%	91.5%	91.0%	91.3%
$10^{-5}$	256	87.4%	87.8%	87.6%	87.7%

*Note:* The best results are shown in bold, and the second-best results are underlined.

**Table 6:** Ablation experiment on spatial feature extractor.

Spatial Feature Extractor	Accuracy	Macro-Precision	Macro-Recall	Macro-F1
FPN	**92.2%**	**92.5%**	**92.4%**	**92.4%**
CNN	90.4%	90.8%	90.8%	90.7%

*Note:* The best results are shown in bold.

**Table 7:** Ablation experiment on temporal feature extractor.

Temporal Feature Extractor	Accuracy	Macro-Precision	Macro-Recall	Macro-F1
Transformer	**92.2%**	**92.5%**	**92.4%**	**92.4%**
GRU	89.3%	89.6%	89.6%	89.5%
LSTM	90.6%	91.2%	90.9%	90.9%

*Note:* The best results are shown in bold.

**Table 8:** Ablation experiment on human trajectory data duration.

Duration	Accuracy	Macro-Precision	Macro-Recall	Macro-F1
15 s	87.2%	87.6%	87.4%	87.4%
30 s	88.7%	89.3%	89.1%	89.0%
60 s	**92.2%**	**92.5%**	**92.4%**	**92.4%**
120 s	92.0%	92.2%	92.2%	92.1%

*Note:* The best results are shown in bold.

**Table 9:** Ablation experiment of floorplan representation.

Floorplan Type	Accuracy	Macro-Precision	Macro-Recall	Macro-F1
None	83.8%	83.9%	84.2%	83.9%
Single-channel	80.5%	81.9%	80.7%	80.7%
Three-channel	**92.2%**	**92.5%**	**92.4%**	**92.4%**

*Note:* The best results are shown in bold.

**Table 10:** Ablation experiment of feature fusion.

Feature Fusion Method	Accuracy	Macro-Precision	Macro-Recall	Macro-F1
Feature Addition	88.2%	88.8%	88.4%	88.4%
Feature Concatenation	89.7%	90.3%	90.0%	90.0%
Attention Mechanism	**92.2%**	**92.5%**	**92.4%**	**92.4%**

*Note:* The best results are shown in bold.

## Data Availability

The original contributions presented in this study are included in the article, further inquiries can be directed to the corresponding author.
